# Warranty period of a zero coronary artery calcium score in Japanese adults

**DOI:** 10.1007/s11604-026-01980-0

**Published:** 2026-03-31

**Authors:** Hidenobu Takagi, Masaharu Hirano, Takashi Asano, Junichi Taguchi, Hideki Ota, Shunji Mugikura, Kei Takase

**Affiliations:** 1 Department of Advanced Radiological Imaging Collaborative Research, Tohoku University, Seiryo, 1-1, Aoba, Sendai, Miyagi 980-8574 Japan; 2 Department of Diagnostic Radiology, Tohoku University Hospital, Sendai, Japan; 3 Department of Early Imaging Diagnosis Joint Research, Tohoku University, Sendai, Japan; 4 Yamanakako Clinic, Minamitsuru, Japan; 5 Tokyo Midtown Clinic, Minato, Tokyo Japan; 6 Medical IT center, Tohoku University Hospital, Sendai, Japan; 7 Division of Image Statistics, Tohoku Medical Megabank Organization, Tohoku University, Sendai, Japan

**Keywords:** Coronary artery calcium, Atherosclerosis, Computed tomography, Prevention

## Abstract

**Background:**

The “warranty period” of a zero coronary artery calcium (CAC) score—the time until conversion to a detectable score—remains undefined for the Japanese population. This study determined the time to CAC incidence in a cohort of asymptomatic Japanese individuals with a CAC score of 0.

**Materials and methods:**

This retrospective study analyzed 1,395 asymptomatic Japanese adults (median age 57 years [range 40–90]; 56% men) with a baseline CAC score of 0 who underwent repeated CAC scanning (median follow-up, 4.1 years). The time to conversion to detectable CAC (> 0) was estimated using Kaplan-Meier analysis and Cox proportional hazards models. Warranty periods were defined as the estimated time to reach cumulative incidence thresholds of 10% and 15%. Continuous relationships between baseline characteristics and warranty periods were visualized using spline curves.

**Results:**

The progression from a zero to a positive CAC score was significantly associated with sex, age, and baseline risk. The estimated time to reach a 10% cumulative incidence was shorter for men (2.5 years; 95% CI, 2.1–3.0) than for women (4.0 years; 95% CI, 3.0–4.6). Interactions were observed between sex and age (*p* = 0.006) and sex and atherosclerotic cardiovascular disease (ASCVD) risk (*p* = 0.001). In men, the warranty period (10% threshold) shortened rapidly with age, decreasing from 3.5 years at age 42 to < 2.0 years at age 68. Conversely, women maintained a period > 3.0 years even at age 68. Higher baseline ASCVD risk scores were associated with shorter warranty periods; for the 10% threshold, the period dropped from 4.1 years at 1% risk to approximately 1.6 years for those with > 20% risk.

**Conclusions:**

The warranty period for a zero CAC score in Japanese individuals varies substantially by age, sex, and cardiovascular risk. Older men and high-risk individuals exhibit rapid conversion (< 2 years), suggesting the need for more frequent surveillance.

**Supplementary Information:**

The online version contains supplementary material available at 10.1007/s11604-026-01980-0.

## Introduction

Atherosclerotic cardiovascular disease (ASCVD) remains a leading cause of morbidity and mortality worldwide [1, 2]. Approximately 50% of ASCVD-related deaths occur in asymptomatic individuals, underscoring the critical need for accurate risk stratification and timely preventive interventions in this population [3]. As the progression of atherosclerosis is largely driven by modifiable risk factors [4], the early identification of subclinical coronary atherosclerosis is essential to initiate interventions that can slow the progression to overt coronary heart disease, such as myocardial infarction.

Coronary artery calcium (CAC), quantified by non-contrast cardiac computed tomography (CT), is a robust marker of subclinical atherosclerosis strongly associated with ASCVD risk [5, 6]. The absence of coronary calcification (i.e., CAC = 0) serves as a powerful negative risk predictor, associated with a very low risk of future coronary events [7–12]. Consequently, the 2019 AHA/ACC guidelines suggest that individuals with intermediate 10-year ASCVD risk can avoid statin therapy if their CAC score is 0 [13].

The prevalence and severity of CAC vary substantially by age, sex, and ethnicity [14, 15]. A significant proportion of Japanese individuals in the target age group for ASCVD risk assessment fall into the intermediate-risk category yet have a CAC score of 0 [15]. For these individuals, determining the appropriate re-assessment interval is crucial for long-term risk management. Defining the time course for CAC development specifically in the Japanese population would help establish evidence-based re-screening guidelines and provide insights into ethnic differences in atherosclerotic progression. Therefore, this study aimed to determine the time to CAC incidence in a large cohort of asymptomatic Japanese individuals with a baseline CAC score of 0.

## Materials and methods

### Study design and cohort

This single-center retrospective observational study was approved by the Ethics Committee of Tohoku University (identifier: 2023-1-855). The requirement for written informed consent was waived due to the retrospective nature of the study.

**Fig. 1:**
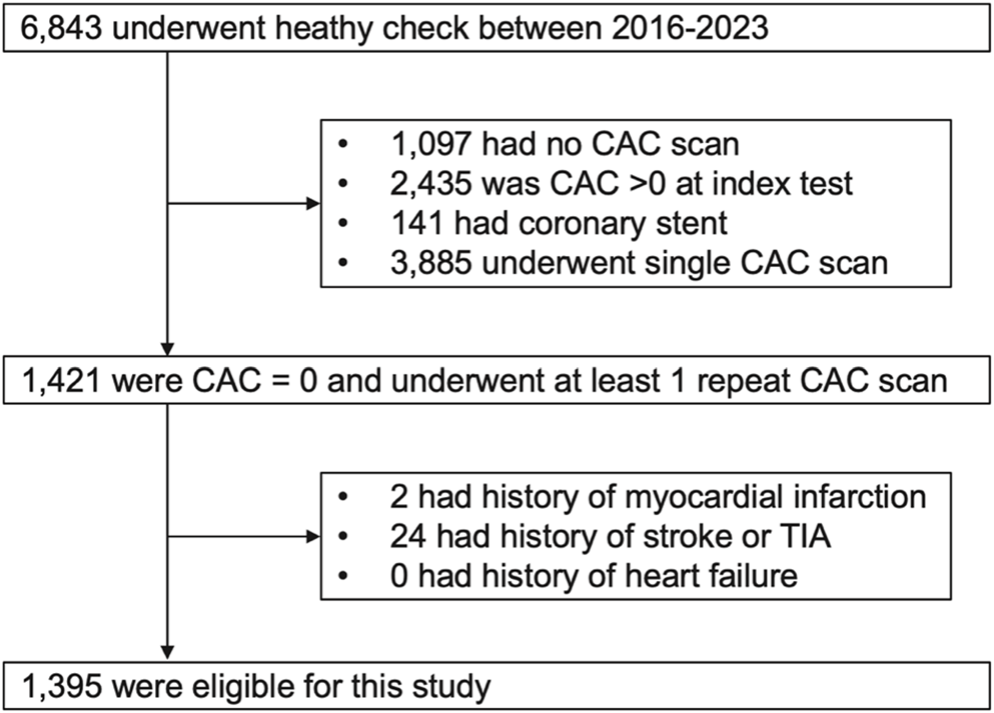
Flowchart of study participant selection. Out of 6,843 individuals screened, 1,395 asymptomatic participants with a baseline CAC score of 0 and at least one follow-up scan were included in the final analysis. Major exclusions were detectable baseline CAC >0, lack of repeat scanning, and history of cardiovascular disease. CAC = coronary artery calcium; TIA = transient ischemic attack

Figure 1 illustrates the study eligibility flow. We included asymptomatic individuals who underwent comprehensive health check-ups at Yamanakako Clinic (Yamanashi, Japan) between 2016 and 2023. CAC scanning was an optional, self-paid component of this program. Repeat CAC scanning was not performed according to a pre-specified study protocol; rather, follow-up data were available only for participants who voluntarily elected to undergo subsequent health check-ups. We excluded individuals who did not undergo CAC scanning (representing those who opted out), those with detectable CAC (score > 0) at baseline, those with only a single CAC scan (no follow-up), and those with a history of percutaneous coronary intervention, myocardial infarction, stroke, transient ischemic attack, or heart failure at baseline.

### Clinical data collection and laboratory measurements

Data on age, sex, medical history, medication use, and smoking status were obtained via a standardized questionnaire and verified against electronic medical records. Obesity was defined as a body mass index ≥ 25 kg/m². Hypertension was defined as systolic blood pressure ≥ 140 mmHg, diastolic blood pressure ≥ 90 mmHg, or current use of antihypertensive medication. Hypercholesterolemia was defined as a total cholesterol level ≥ 220 mg/dL. These definitions align with those used in the Hisayama Study [16]. The 10-year atherosclerotic cardiovascular disease (ASCVD) risk was calculated using a validated prediction model derived from the Hisayama Study [17]. Risk categories were classified as low (< 5.0%), intermediate (5.0% to 19.9%), and high (≥ 20%), consistent with American guidelines [13].

### Coronary artery calcium scanning and measurements

Baseline and follow-up CAC scans were performed using a standardized protocol on a 32-slice CT scanner (Biograph mCT Flow, Siemens Healthineers, Erlangen, Germany) without iodine contrast and with electrocardiography gating. Scan parameters included a tube voltage of 120 kVp, tube current regulated by automatic exposure control (CARE Dose 4D), and a slice thickness and increment of 3.0 mm. CAC was quantified by experienced technicians using semi-automated software (AZE Virtual Place; AZE, Inc, Tokyo, Japan) according to the Agatston method [18].

### Statistical analyses

Continuous variables are presented as medians with interquartile ranges (Q1–Q3), and categorical variables as numbers and percentages. To describe the overall progression of coronary calcification, we estimated the cumulative incidence of conversion from a baseline CAC score of 0 to scores > 0, >10, and > 100 using the Kaplan-Meier method. For individuals with multiple follow-up scans, the time-to-event for each specific threshold was defined as the time to the first scan demonstrating that the respective threshold (> 0, > 10, or > 100) was reached. Subsequent scans after reaching a specific threshold were not included in the analysis for that endpoint. Participants who did not reach the respective threshold during the study period were censored at the time of their last available scan. To assess the impact of demographics and risk profiles, we stratified the cumulative incidence of detectable CAC (score > 0) by age group (< 55, 55–65, > 65 years) and 10-year ASCVD risk category (Low < 5%, Intermediate 5–19.9%, High ≥ 20%), assessing differences with the log-rank test.

To determine the “warranty period” for a zero CAC score, we estimated the time required for the cumulative incidence of detectable CAC to reach clinically relevant thresholds of 10% (number needed to scan [NNS] = 10), 15% (NNS = 6), and 20% (NNS = 5). Before estimation, we confirmed the validity of the Cox proportional hazards models used for these projections by testing the proportional hazards assumption using Schoenfeld residuals. We then constructed Cox proportional hazards models with age and sex, or the 10-year ASCVD risk score, as covariates. Based on these models, we generated predicted survival curves for a continuous sequence of baseline ages and 10-year ASCVD risk scores. From each predicted curve, the time point at which the cumulative incidence reached the specified threshold was extracted as the point estimate. Corresponding 95% confidence intervals (CIs) were derived from the intersection of the threshold with the upper and lower 95% confidence bounds of the predicted survival curves. To visualize the continuous relationship between baseline characteristics and the warranty period, we applied locally weighted scatterplot smoothing (LOWESS) regression with a span of 0.7 to smooth both the point estimates and their 95% CIs.

As a sensitivity analysis, we performed the same warranty period estimation using a Weibull parametric survival model to confirm the robustness of our findings [19–21]. To evaluate potential selection bias due to loss to follow-up, we compared the baseline characteristics of included participants (those with repeat CAC scanning) versus those excluded due to a lack of follow-up scans. Differences between groups were assessed using the Wilcoxon rank-sum test for continuous variables and the Chi-square test for categorical variables. A two-sided p-value < 0.05 was considered statistically significant. All analyses were performed using R version 4.4.0 [22].

## Results

### Participant characteristics

Of 6,843 individuals screened, 1,395 asymptomatic participants with a baseline CAC score of 0 were included in the final analysis (Fig. 1). Primary exclusions were the absence of CAC testing, detectable baseline CAC (> 0), lack of repeat scanning, or history of cardiovascular disease. Table 1 summarizes baseline characteristics. The median age was 57 years (IQR 50–64), and 56% were men. Hypertension (35%) and hypercholesteremia (50%) were common, while diabetes mellitus was rare (4.7%). Most participants fell into the low (34%) or intermediate (61%) 10-year ASCVD risk categories. The risk factor profiles differed notably between sexes; men had a higher prevalence of hypertension, hypercholesteremia, and smoking, as well as higher 10-year ASCVD risk scores compared to women. The median follow-up duration was 4.1 years (IQR 2.0–6.0). Regarding potential selection bias, participants excluded due to a lack of follow-up scans (*n* = 1,713) were significantly younger and had lower antihypertensive medication use but higher smoking rates compared to the study cohort (Supplemental Table 1). However, the median 10-year ASCVD risk score was identical (5.6%) between the groups, suggesting a broadly comparable cardiovascular risk profile.

**Table 1:** Characteristics of the study population

Variables	Overall *N* = 1,395	Men *N* = 784	Women *N* = 611
Age, yr.	57 (50–64)	53 (47–61)	59 (55–66)
Age categories, n (%)			
<55 years	590 (42%)	443 (57%)	147 (24%)
55–65 years	510 (37%)	223 (28%)	287 (47%)
>65 years	295 (21%)	118 (15%)	177 (29%)
Body mass index, kg/m^2^	23.6 (21.4–25.7)	24.6 (22.8–26.8)	21.7 (19.8–24.1)
Waist circumference, cm	89 (83–96)	91 (86–97)	85 (79–93)
Obesity, n (%)	455 (33%)	351 (45%)	104 (17%)
Hypertension, n (%)	487 (35%)	306 (39%)	181 (30%)
Hypercholesteremia, n (%)	696 (50%)	353 (45%)	343 (56%)
Diabetes mellitus, n (%)	66 (4.7%)	52 (6.7%)	14 (2.3%)
Systolic blood pressure, mmHg	125 (113–135)	126 (115–135)	124 (112–135)
Diastolic blood pressure, mmHg	78 (70–86)	80 (73–88)	75 (67–82)
Serum total cholesterol, mg/dL	220 (197–244)	215 (190–240)	226 (204–250)
Serum LDL cholesterol, mg/dL	130 (108–150)	128 (107–149)	131 (110–152)
Serum HDL cholesterol, mg/dL	64 (53–77)	57 (48–68)	73 (62–87)
Serum triglycerides, mg/dL	96 (70–143)	116 (81–173)	81 (61–106)
Hemoglobin A1c, %	5.1 (4.9–5.4)	5.1 (4.9–5.4)	5.1 (5.0–5.3)
Current or past smoker, n (%)	692 (50%)	550 (60%)	142 (23%)
Antihypertensive agents, n (%)	261 (19%)	162 (21%)	99 (16%)
Statin, n (%)	144 (10%)	72 (9.2%)	72 (12%)
10-year ASCVD risk, %	5.6 (4.2–9.8)	7.4 (4.2–12.8)	5.6 (4.2–7.4)
Risk categories, n (%)			
Low (< 5%)	479 (34%)	206 (26%)	273 (45%)
Intermediate (5%–19.9%)	849 (61%)	520 (66%)	329 (54%)
High (≥ 20%)	67 (4.8%)	58 (7.4%)	9 (1.5%)

Continuous variables are summarized as median and interquartile ranges (25–75%) in parentheses, and categorical variables are summarized raw number and percentages in parentheses

LDL = low-density lipoprotein; HDL = high-density lipoprotein; ASCVD = atherosclerotic cardiovascular disease

### Cumulative incidence of coronary artery calcium

**Fig. 2:**
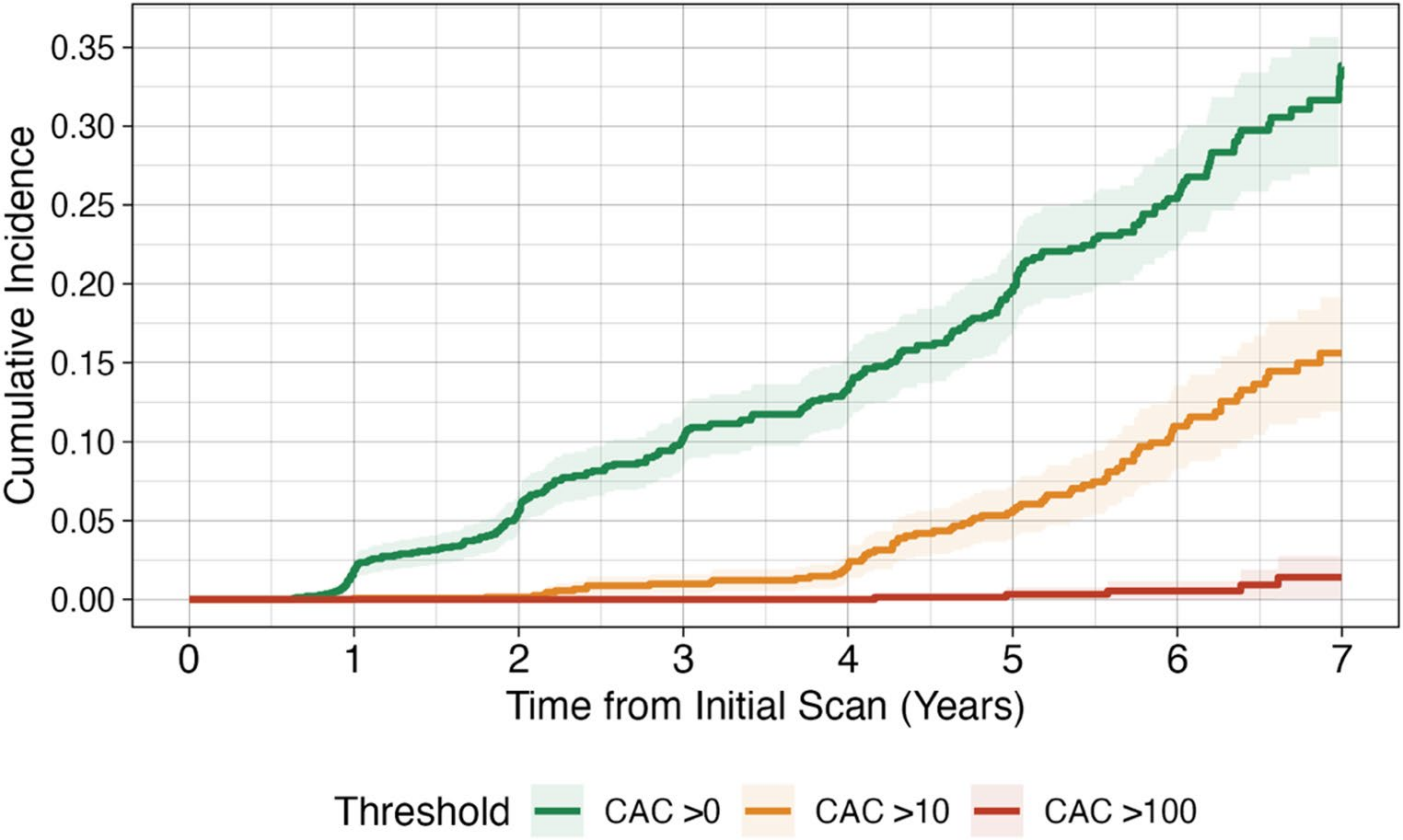
Cumulative incidence of coronary artery calcium progression. The curves illustrate the cumulative incidence of developing detectable CAC (score >0), mild or more CAC (score >10), and moderate or more CAC (score >100) among asymptomatic Japanese with a baseline CAC score of 0. Shaded areas indicate 95% confidence intervals. CAC = coronary artery calcium

**Fig. 3:**
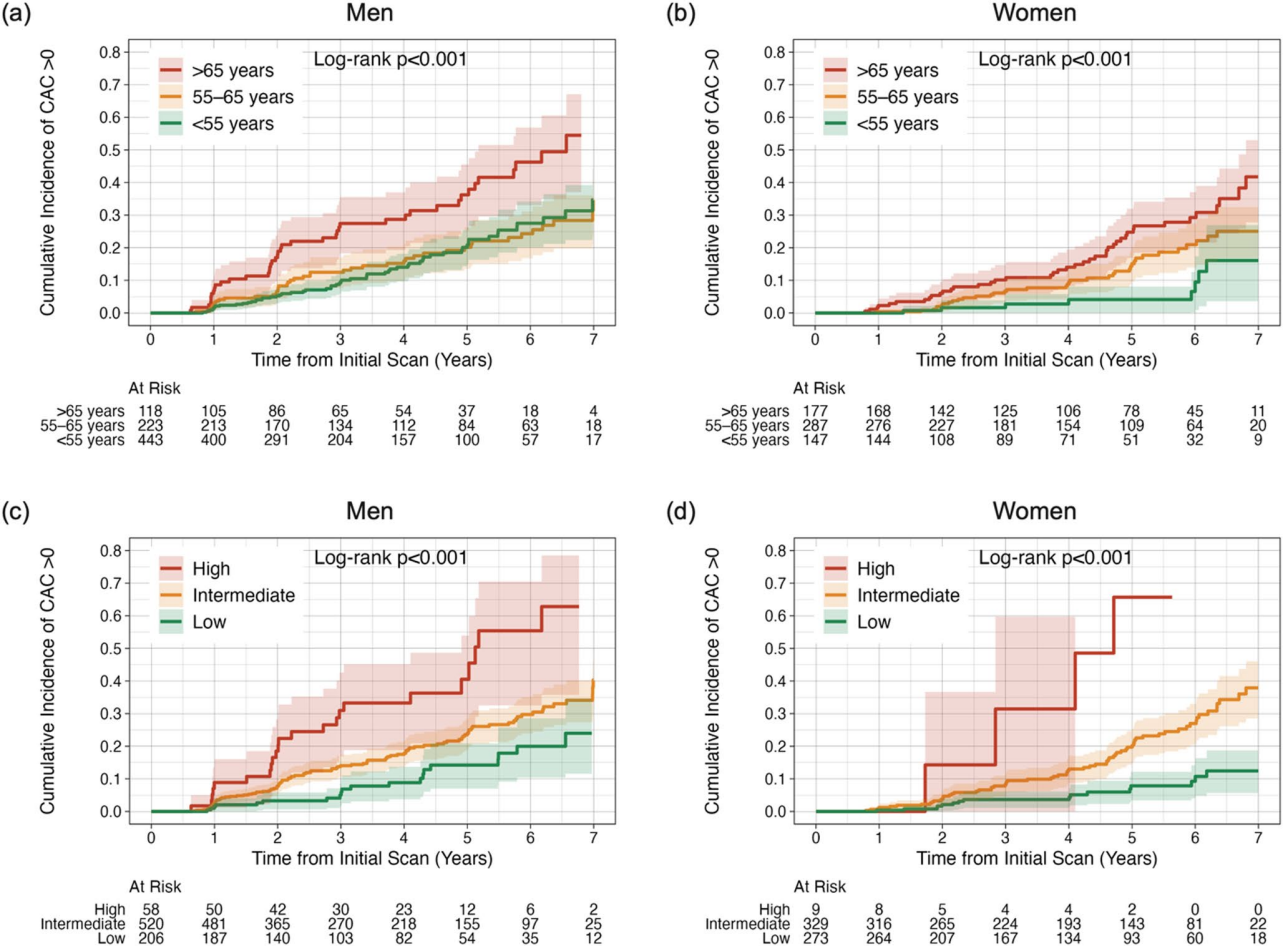
Cumulative incidence of detectable coronary artery calcium according to age and ASCVD risk. Kaplan-Meier curves show the cumulative incidence of developing detectable CAC (score > 0). Panel **(a)** and **(b)** display stratification by age groups (< 55, 55–65, and > 65 years) for men and women, respectively. Panels **(c)** and **(d)** display stratification by 10-year ASCVD risk categories (Low < 5%, Intermediate 5–19.9%, and High ≥ 20%). Shaded regions indicate 95% confidence intervals. ASCVD = atherosclerotic cardiovascular disease; CAC = coronary artery calcium

The number of rescreened participants decreased over time, from 997 (71%) at 0–2 years to 279 (20%) after 6 years (Table 2). The crude incidence of detectable CAC (score > 0) increased from 7.2% at 0–2 years to 11.5% at 4–6 years. Figure 2 illustrates incidence stratified by severity. While the cumulative incidence of detectable CAC increased steadily, progression to moderate CAC (> 100) remained negligible (< 1%) during the first 4 years. Stratified analyses showed significant differences in CAC incidence by sex, age, and ASCVD risk (all Log-rank *p* < 0.001) (Supplemental Fig. 1). As shown in Fig. 3, incidence rates increased with advancing age and higher risk categories in both sexes. Notably, men in the high-risk group (≥ 20%) exhibited a rapid rise, exceeding 50% cumulative incidence by year 6.

**Table 2:** Number of participants scanned

Time from index CAC Scan	Number of participants rescanned with baseline CAC = 0	Number of participants with incident CAC *N* (% of those rescanned)
0–2 yrs.	997 (71%)	72 (7.2%)
2–4 yrs.	864 (61%)	71 (8.2%)
4–6 yrs.	620 (44%)	71 (11.5%)
> 6 yrs.	279 (20%)	28 (10.0%)

Participants with multiple follow-up scans are counted in each applicable time interval

### Estimated time to onset of coronary artery calcium (warranty period)

**Fig. 4:**
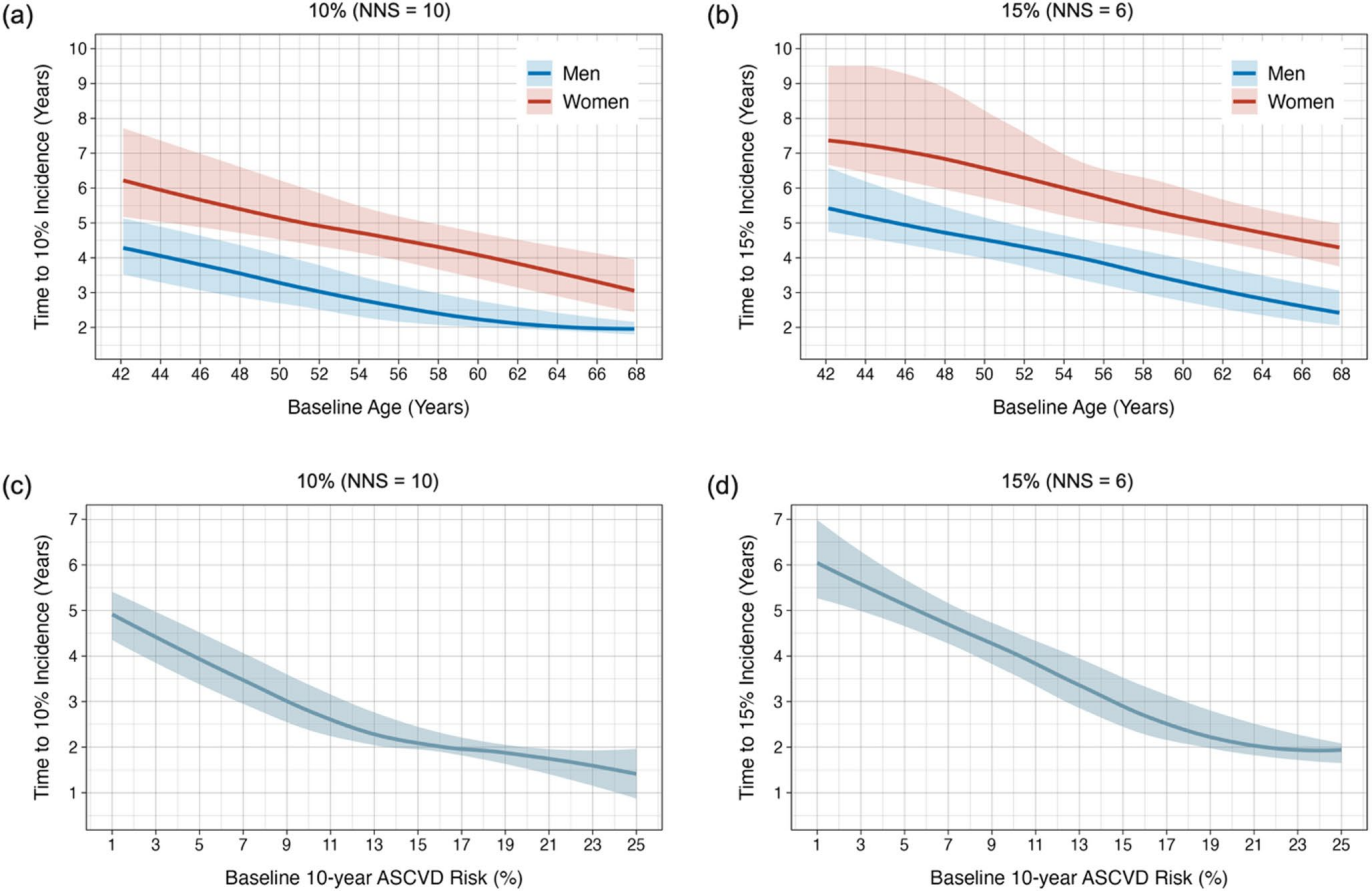
Estimated Time to onset of detectable coronary artery calcium according to age and ASCVD risk by cox proportional hazard model. The spline curves illustrate the estimated years until the incidence of detectable CAC (score > 0) reaches 10% (NNS = 10; **a**, **c**) and 15% (NNS = 6; **b**, **d**). Panels **(a)** and **(b)** display estimates stratified by baseline age and sex. Panels **(c)** and **(d)** display estimates according to the baseline 10-year ASCVD risk score. Shaded regions indicate 95% confidence intervals. ASCVD = atherosclerotic cardiovascular disease; CAC = coronary artery calcium; NNS = number need to scan

The proportional hazards assumption was satisfied for all models (global *p* > 0.05 for all). Table 3; Fig. 4 present the estimated time to reach 10% and 15% CAC incidence. Overall, the time to 10% incidence was significantly shorter for men (2.5 years; 95% CI, 2.1–3.0) than for women (4.0 years; 95% CI, 3.0–4.6). Similarly, for the 15% threshold, men required 3.8 years compared to 5.0 years for women. Significant interactions were observed between sex and age (*p* = 0.006) and between sex and ASCVD risk (*p* = 0.001). Spline curves in Fig. 4 visualize these continuous relationships. In men, the estimated time to 10% incidence decreased from approximately 4.2 years at age 42 to 1.9 years at age 68. Women maintained longer intervals, decreasing from approximately 6.2 years to 2.9 years over the same age range. Regarding ASCVD risk, the warranty period shortened non-linearly as risk scores increased; for the 10% threshold, the time dropped from 4.9 years at 1% risk to approximately 1.8 years at > 20% risk (Fig. 4c). A similar pattern was observed for the 15% threshold (Fig. 4d), where the warranty period decreased from 6 years in the lowest risk range to approximately 2 years in the highest risk range.

**Table 3:** Time to incidence of CAC by cox proportional hazard model

	10% (NNS = 10) Men	10% (NNS = 10) Women	15% (NNS = 6) Men	15% (NNS = 6) Women	20% (NNS = 5) Men	20% (NNS = 5) Women
All	2.5 (2.1–3.0)	4.0 (3.0–4.6)	3.8 (3.0–4.3)	5.0 (4.4–5.8)	4.6 (4.1–5.0)	6.0 (5.1–6.8)
*Age category*						
<55 years	3.0 (2.5–4.0)	4.9 (4.1–6.2)	4.3 (3.8–5.0)	6.2 (5.1–7.5)	5.0 (4.6–6.1)	7.1 (6.3–NR)
55–65 years	2.8 (2.2–3.7)	4.6 (3.9–5.3)	4.0 (3.0–4.8)	5.8 (5.0–7.0)	4.8 (4.1–5.7)	6.8 (6.0–NR)
>65 years	1.9 (1.6–2.1)	2.5 (2.1–3.4)	2.1 (2.0–2.9)	3.8 (3.0–4.6)	2.9 (2.2–3.8)	4.7 (4.0–5.4)
*Risk category*						
Low	4.7 (4.0–6.0)	5.3 (4.6–7.0)	5.9 (5.0–7.3)	6.8 (5.9–NR)	7.0 (6.0–NR)	7.4 (7.0–NR)
Intermediate	2.4 (2.1–3.0)	3.0 (2.4–4.0)	3.7 (3.0–4.2)	4.3 (3.8–5.0)	4.4 (4.0–5.0)	5.0 (4.6–6.0)
High	1.6 (1.0–2.0)	1.9 (1.4–2.5)	2.0 (1.7–2.6)	2.2 (1.9–3.8)	2.2 (2.0–3.4)	2.9 (2.1–4.6)

NNS = number need to scan; NR = not reached

### Sensitivity analyses

Estimates derived from the Weibull parametric survival model were consistent with the primary Cox proportional hazards analysis (Supplemental Table 2). For instance, the time to 10% incidence in men was estimated at 2.8 years (95% CI, 2.5–3.1) using the Weibull model, compared to 2.5 years in the primary analysis. The trajectories of the age- and risk-dependent curves were also similar between models (Supplemental Fig. 2), confirming the robustness of the findings.

## Discussion

In this large-scale longitudinal study of asymptomatic Japanese individuals with a baseline CAC score of 0, we determined the “warranty period” (time to CAC incidence) stratified by age, sex, and ASCVD risk. Our main findings are threefold. First, the warranty period shortened significantly with advancing age, with men developing calcification markedly earlier than women across all age groups. Second, a higher baseline 10-year ASCVD risk score was strongly associated with a shorter warranty period in a dose-response manner. Third, we established specific time intervals required for the cumulative incidence of detectable CAC to reach clinically relevant thresholds of 10% (NNS = 10) and 15% (NNS = 6). These estimates provide the first population-specific reference tool to guide personalized re-screening intervals for the Japanese population. Additionally, we observed a substantial lag time between the onset of detectable CAC and the progression to more advanced calcification (CAC > 100), suggesting a window of opportunity for early intervention.

The observed sex-dependent disparity in the time to CAC incidence aligns with established biological mechanisms and is strongly corroborated by our recent cross-sectional analysis of the same cohort [15]. In that study, the prevalence of detectable CAC in men surged rapidly between the ages of 40 and 60, whereas in women, a comparable increase was delayed until after the age of 60. The present longitudinal study extends these observations by quantifying the “speed” of this progression. The rapid shortening of the warranty period in middle-aged men directly corresponds to the steep rise in prevalence seen in the cross-sectional data, reflecting an accelerated atherosclerotic process during this life stage. Conversely, the sustained warranty period in pre- and peri-menopausal women mirrors the delayed onset of calcification prevalence, likely attributable to the vasoprotective effects of estrogen [23]. Together, these complementary datasets provide a comprehensive picture of CAC dynamics in the Japanese population: men experience an earlier “calcification phase” starting in their 40s, while women benefit from a prolonged “latent phase” that extends well into their 60s.

Our warranty period estimates also highlight important ethnic differences when compared to the MESA study [20]. In our Japanese cohort, the estimated times to 15% (NNS = 6) and 20% (NNS = 5) CAC incidence for men were 3.8 and 4.6 years, respectively. These intervals are longer than those reported for White and Black men in MESA but shorter than those for Chinese American men. Conversely, for Japanese women, the warranty periods were 5.0 and 6.0 years, respectively, which are longer even than those of Chinese American women in MESA. These variations likely stem from a combination of factors, including differences in ethnicity, lifestyle-related risk profiles at baseline (Supplemental Table 3), and potentially the high follow-up rate in the early phase of our study. Specifically, the markedly longer warranty period in Japanese women suggests a lower atherosclerotic burden in this subgroup, reinforcing the need for population-specific screening guidelines rather than applying Western or even generalized Asian data.

The robust inverse association between baseline 10-year ASCVD risk scores and the warranty period provide compelling radiographic evidence that a higher burden of cardiovascular risk factors accelerates subclinical vascular damage. Our data demonstrate a clear dose-response relationship: as the ASCVD risk score increases, the time to developing calcification shortens progressively. This non-linear decay is particularly steep in the transition from low to intermediate risk, highlighting that even moderate elevations in risk profile can significantly precipitate the onset of calcification. These findings are consistent with observations from the MESA study, which also reported a graded shortening of the warranty period with increasing risk factor burden and demonstrated the varying contributions of individual risk factors such as diabetes and smoking to CAC incidence [24]. Importantly, our results extend this concept to the Japanese population, showing that despite a generally lower atherosclerotic baseline, the fundamental biological impact of aggregated risk factors on arterial calcification remains consistent. This underscores the potential of using the ASCVD risk score not only for estimating event risk but also for tailoring the timing of initial and repeat CAC screening; individuals with intermediate-to-high risk scores warrant earlier and more frequent surveillance to detect the transition from a “zero” to a “positive” score. While the emergence of mild calcification (CAC > 0) does not imply an immediate high risk of cardiovascular events, it signifies the loss of the unique “very low risk” status (“power of zero”) that justifies withholding statin therapy. According to the 2019 AHA/ACC guidelines [13], for adults with intermediate (10-year ASCVD risk 7.5% to 20%) or borderline (5% to 7.5%) risk, initiating statin therapy is reasonable if the CAC score is 1 to 99 (particularly for those ≥ 55 years of age) or ≥ 100 (or ≥ 75th percentile). Although these specific US-based criteria may not be directly applicable to Japanese clinical practice in their entirety, they reinforce the concept that the transition to a positive CAC score marks a critical clinical inflection point. This transition signals the need to re-evaluate preventive strategies—such as considering statin initiation or intensifying lifestyle modifications—based on the individual’s comprehensive ASCVD risk profile.

This study has several limitations. First, this was a single-center, retrospective observational study of voluntary health check-up participants at a facility requiring a membership fee. This design introduces potential selection bias, as participants may have higher socioeconomic status and health awareness compared to the general population. Additionally, our analysis comparing included participants with those excluded due to a lack of follow-up (Supplemental Table 1) showed that the excluded group was significantly younger. These factors suggest that our warranty period estimates may more closely represent a slightly older, health-conscious population with higher socioeconomic status and might not be fully generalizable to the general public or younger individuals who do not undergo regular health check-ups. Second, we defined the warranty period based on CAC conversion, not hard cardiovascular events. Since we did not evaluate the occurrence of outcomes such as myocardial infarction or cardiovascular death, the warranty period defined here represents the duration of maintaining a zero CAC score and cannot be directly equated to a period free from clinical cardiovascular events. Future prospective studies are needed to confirm the prognostic validity of these intervals in the Japanese population. Third, non-contrast CT cannot detect non-calcified plaque; thus, a zero score does not rule out all atherosclerosis. Fourth, due to the relatively short follow-up (median 4.1 years), we could not fully estimate warranty periods for advanced calcification endpoints or higher incidence thresholds (e.g., 20%) in low-risk subgroups. Fifth, because health check-ups were performed at discrete intervals rather than continuously, the data are subject to interval censoring, meaning the precise moment of CAC onset could not be determined. Finally, the number of participants in certain subgroups (e.g., high-risk young women) was small, limiting estimation precision.

In conclusion, we determined the time to the incidence of CAC in asymptomatic Japanese individuals with a baseline CAC score of 0, stratified by age, sex, and ASCVD risk. Our findings provide compelling radiographic evidence of the cumulative vascular damage associated with aging, male sex, and elevated cardiovascular risk factors. Clinically, these population-specific warranty periods provide a practical framework for determining re-screening intervals when follow-up testing is considered appropriate, facilitating more personalized risk assessment strategies tailored to the specific profile of each patient.

## Supplementary Information

Below is the link to the electronic supplementary material.

**Supplementary Material**.

## Data Availability

The data that support the findings of this study are not publicly available due to ethical restrictions protecting patient privacy. De-identified data underlying the results presented in this article (including tables, figures, and supplemental materials) may be made available from the corresponding author upon reasonable request. Any request will require a formal data use agreement and approval from the Institutional Review Board of Tohoku University before data can be shared.

## Abbreviations

ASCVD: Atherosclerotic cardiovascular disease
CAC: Coronary artery calcium
CI: Confidence interval
CT: Computed tomography
NNS: Number need to scan

## References

[1] Zhang Y, Deng Q, Hu P, Zeng Q, Li B, Liu J. Burden and risk factors of cardiovascular diseases in Asia, 1990–2021: An analysis for GBD 2021. JACC Asia [Internet]. 2025; Available from: https://linkinghub.elsevier.com/retrieve/pii/S277237472500518610.1016/j.jacasi.2025.08.02441201423

[2] Global Burden of Cardiovascular Diseases and Risks. 2023 Collaborators. Global, regional, and national burden of cardiovascular diseases and risk factors in 204 countries and territories, 1990–2023. J Am Coll Cardiol. 2025;86:2167–243. 10.1016/j.jacc.2025.08.015 40990886

[3] Greenland P, Alpert JS, Beller GA, Benjamin EJ, Budoff MJ, Fayad ZA, et al. 2010 ACCF/AHA guideline for assessment of cardiovascular risk in asymptomatic adults: a report of the American College of Cardiology Foundation/American Heart Association Task Force on Practice Guidelines. J Am Coll Cardiol. 2010;56:e50–103. 21144964 10.1016/j.jacc.2010.09.001

[4] D’Agostino RB, Sr, Vasan RS, Pencina MJ, Wolf PA, Cobain M, Massaro JM, et al. General cardiovascular risk profile for use in primary care: the Framingham Heart Study: The Framingham heart study. Circulation. 2008;117:743–53. 18212285 10.1161/CIRCULATIONAHA.107.699579

[5] Detrano R, Guerci AD, Carr JJ, Bild DE, Burke G, Folsom AR, et al. Coronary calcium as a predictor of coronary events in four racial or ethnic groups. N Engl J Med. 2008;358:1336–45. 18367736 10.1056/NEJMoa072100

[6] Budoff MJ, Young R, Burke G, Jeffrey Carr J, Detrano RC, Folsom AR, et al. Ten-year association of coronary artery calcium with atherosclerotic cardiovascular disease (ASCVD) events: the multi-ethnic study of atherosclerosis (MESA). Eur Heart J. 2018;39:2401–8. 29688297 10.1093/eurheartj/ehy217 PMC6030975

[7] Mieres JH, Shaw LJ, Arai A, Budoff MJ, Flamm SD, Hundley WG, et al. Role of noninvasive testing in the clinical evaluation of women with suspected coronary artery disease: Consensus statement from the Cardiac Imaging Committee, Council on Clinical Cardiology, and the Cardiovascular Imaging and Intervention Committee, Council on Cardiovascular Radiology and Intervention, American Heart Association: Consensus statement from the cardiac imaging committee, council on clinical cardiology, and the cardiovascular imaging and intervention committee, council on cardiovascular radiology and intervention, American heart association. Circulation. 2005;111:682–96. 15687114 10.1161/01.CIR.0000155233.67287.60

[8] Valenti V, Ó Hartaigh B, Heo R, Cho I, Schulman-Marcus J, Gransar H, et al. A 15-year warranty period for asymptomatic individuals without coronary artery calcium: a prospective follow-up of 9,715 individuals. JACC Cardiovasc Imaging. 2015;8:900–9. 26189116 10.1016/j.jcmg.2015.01.025 PMC4537357

[9] Blaha MJ, Cainzos-Achirica M, Greenland P, McEvoy JW, Blankstein R, Budoff MJ, et al. Role of coronary artery calcium score of zero and other negative risk markers for cardiovascular disease: The Multi-Ethnic Study of atherosclerosis (MESA): The Multi-Ethnic Study of atherosclerosis (MESA). Circulation. 2016;133:849–58. 26801055 10.1161/CIRCULATIONAHA.115.018524 PMC4775391

[10] Mortensen MB, Fuster V, Muntendam P, Mehran R, Baber U, Sartori S, et al. Negative risk markers for cardiovascular events in the elderly. J Am Coll Cardiol. 2019;74:1–11. 31272534 10.1016/j.jacc.2019.04.049

[11] Nasir K, Cainzos-Achirica M. Role of coronary artery calcium score in the primary prevention of cardiovascular disease. BMJ. 2021;373:n776. 33947652 10.1136/bmj.n776

[12] Agha AM, Pacor J, Grandhi GR, Mszar R, Khan SU, Parikh R, et al. The prognostic value of CAC zero among individuals presenting with chest pain: a meta-analysis. JACC Cardiovasc Imaging. 2022;15:1745–57. 36202453 10.1016/j.jcmg.2022.03.031

[13] Arnett DK, Blumenthal RS, Albert MA, Buroker AB, Goldberger ZD, Hahn EJ et al. 2019 ACC/AHA guideline on the primary prevention of cardiovascular disease: a report of the American college of cardiology/American heart association task force on clinical practice guidelines. Circulation. 2019;140. 10.1161/CIR.0000000000000678 PMC7734661 30879355

[14] de Ronde MWJ, Khoshiwal A, Planken RN, Boekholdt SM, Biemond M, Budoff MJ, et al. A pooled-analysis of age and sex based coronary artery calcium scores percentiles. J Cardiovasc Comput Tomogr. 2020;14:414–20. 32019722 10.1016/j.jcct.2020.01.006

[15] Takagi H, Hirano M, Asano T, Nishimiya K, Obara T, Ota H et al. Age- and sex-specific distribution and reference values of coronary artery calcium in a large asymptomatic Japanese cohort. J Am Heart Assoc. 2026. 10.1161/JAHA.125.046403 10.1161/JAHA.125.046403 PMC13055732 41676943

[16] Hata J, Ninomiya T, Hirakawa Y, Nagata M, Mukai N, Gotoh S, et al. Secular trends in cardiovascular disease and its risk factors in Japanese: half-century data from the Hisayama Study (1961–2009): Half-century data from the Hisayama study (1961–2009). Circulation. 2013;128:1198–205. 23902756 10.1161/CIRCULATIONAHA.113.002424

[17] Arima H, Yonemoto K, Doi Y, Ninomiya T, Hata J, Tanizaki Y, et al. Development and validation of a cardiovascular risk prediction model for Japanese: the Hisayama study. Hypertens Res. 2009;32:1119–22. 19763133 10.1038/hr.2009.161

[18] Agatston AS, Janowitz WR, Hildner FJ, Zusmer NR, Viamonte M Jr, Detrano R. Quantification of coronary artery calcium using ultrafast computed tomography. J Am Coll Cardiol. 1990;15:827–32. 2407762 10.1016/0735-1097(90)90282-t

[19] Dzaye O, Dardari ZA, Cainzos-Achirica M, Blankstein R, Szklo M, Budoff MJ, et al. Incidence of new coronary calcification: time to conversion from CAC = 0. J Am Coll Cardiol. 2020;75:1610–3. 32241379 10.1016/j.jacc.2020.01.047 PMC11698271

[20] Dzaye O, Dardari ZA, Cainzos-Achirica M, Blankstein R, Agatston AS, Duebgen M, et al. Warranty period of a calcium score of zero: comprehensive analysis from MESA. JACC Cardiovasc Imaging. 2021;14:990–1002. 33129734 10.1016/j.jcmg.2020.06.048 PMC8076346

[21] Ichikawa K, Lim J, McClelland RL, Susarla S, Krishnan S, Benzing T, et al. Impact of nonalcoholic hepatic steatosis on the warranty period of a coronary artery calcium score of 0: results from the Multi-Ethnic Study of Atherosclerosis. Circ Cardiovasc Imaging. 2024;17:e016465. 39288206 10.1161/CIRCIMAGING.123.016465 PMC11410342

[22] R Core Team. The R Project for Statistical Computing [Internet]. The R Project for Statistical Computing. [cited 2024 Nov 11]. Available from: https://www.r-project.org/

[23] Mendelsohn ME, Karas RH. The protective effects of estrogen on the cardiovascular system. N Engl J Med. 1999;340:1801–11. 10362825 10.1056/NEJM199906103402306

[24] Dzaye O, Razavi AC, Dardari ZA, Shaw LJ, Berman DS, Budoff MJ, et al. Modeling the recommended age for initiating coronary artery calcium testing among at-risk young adults. J Am Coll Cardiol. 2021;78:1573–83. 34649694 10.1016/j.jacc.2021.08.019 PMC9074911

