# Supplementary material for: Warranty period of a zero coronary artery calcium score in Japanese adults
Source: Jpn J Radiol. 2026 Mar 31;44(7):1150–8. doi: 10.1007/s11604-026-01980-0 (PMC13315475; doi:10.1007/s11604-026-01980-0)
Supplement: Supplementary file 1 — Supplementary Material. [file 11604_2026_1980_MOESM1_ESM.docx]

**Supplemental Materials**

Warranty Period of a Zero Coronary Artery Calcium Score in Japanese Adults

**Supplemental Table 1.** Comparison with Individuals without CAC who Underwent Single CAC Scan

| **Variables** | **Included**  **(Rescanned)**  N = 1,395 | **Excluded**  **(Single Scan)** N = 1,713 | ***P* value** |
| --- | --- | --- | --- |
| Age, yr. | 57 (50–64) | 54 (48–61) | **<0.001** |
| Age categories, n (%) |  |  | **<0.001** |
| <55 years | 590 (42%) | 916 (53%) |  |
| 55–65 years | 510 (37%) | 548 (32%) |  |
| >65 years | 295 (21%) | 249 (15%) |  |
| Women, n (%) | 611 (44%) | 711 (42%) | 0.198 |
| Body mass index, kg/m^2^ | 23.6 (21.4–25.7) | 23.3 (21.0–25.6) | 0.082 |
| Waist circumference, cm | 89 (83–96) | 87 (81–94) | **<0.001** |
| Obesity, n (%) | 455 (33%) | 546 (32%) | 0.659 |
| Hypertension, n (%) | 487 (35%) | 556 (32%) | 0.150 |
| Hypercholesteremia, n (%) | 696 (50%) | 864 (50%) | 0.762 |
| Diabetes mellitus, n (%) | 66 (4.7%) | 61 (3.6%) | 0.258 |
| Systolic blood pressure, mmHg | 125 (113–135) | 126 (115–135) | 0.616 |
| Diastolic blood pressure, mmHg | 78 (70–86) | 80 (72–86) | **<0.001** |
| Serum total cholesterol, mg/dL | 220 (197–244) | 220 (197–245) | 0.499 |
| Serum LDL cholesterol, mg/dL | 130 (108–150) | 130 (110–151) | 0.408 |
| Serum HDL cholesterol, mg/dL | 64 (53–77) | 65 (54–78) | 0.163 |
| Serum triglycerides, mg/dL | 96 (70–143) | 94 (66–139) | **0.039** |
| Hemoglobin A1c, % | 5.1 (4.9–5.4) | 5.1 (4.9–5.3) | **<0.001** |
| Current or past smoker, n (%) | 692 (50%) | 944 (56%) | **0.002** |
| Antihypertensive agents, n (%) | 261 (19%) | 242 (14%) | **<0.001** |
| Statin, n (%) | 144 (10%) | 159 (9.3%) | 0.331 |
| 10-year ASCVD risk, % | 5.6 (4.2–9.8) | 5.6 (4.2–9.8) | **<0.001** |
| Risk categories, n (%) |  |  | **0.010** |
| Low risk (<5%) | 479 (34%) | 662 (39%) |  |
| Intermediate risk (5%–19.9%) | 849 (61%) | 994 (58%) |  |
| High risk (≥20%) | 67 (4.8%) | 57 (3.3%) |  |

Note. — continuous variables are summarized as median and interquartile ranges (25%–75%) in parentheses, and categorical variables are summarized raw number and percentages in parentheses.

LDL = low-density lipoprotein; HDL = high-density lipoprotein; CAC = coronary artery calcium; ASCVD = = atherosclerotic cardiovascular disease.

**Supplemental Table 2**. Time to Incidence of CAC by Weibull Model

|  | **10%**  **(NNS = 10)**  **Men** | **10%**  **(NNS = 10)**  **Women** | **15%**  **(NNS = 6)**  **Men** | **15%**  **(NNS = 6)**  **Women** | **20%**  **(NNS = 5)**  **Men** | **20%**  **(NNS = 5)**  **Women** |
| --- | --- | --- | --- | --- | --- | --- |
| **All** | 2.8 (2.5–3.1) | 3.7 (3.3–4.2) | 3.6 (3.3–4.0) | 4.8 (4.2–5.4) | 4.3 (3.9–4.7) | 5.8 (5.1–6.5) |
| **Age category** |  |  |  |  |  |  |
| <55 years | 3.2 (2.8–3.7) | 4.7 (3.9–5.7) | 4.1 (3.6–4.8) | 6.1 (5.0–7.3) | 5.0 (4.3–5.7) | 7.3 (6.0–8.8) |
| 55–65 years | 2.9 (2.5–3.4) | 4.3 (3.6–5.0) | 3.8 (3.2–4.3) | 5.5 (4.7–6.4) | 4.5 (3.9–5.2) | 6.6 (5.6–7.8) |
| >65 years | 2.0 (1.6–2.3) | 2.9 (2.5–3.4) | 2.5 (2.1–3.0) | 3.7 (3.2–4.3) | 3.0 (2.6–3.5) | 4.5 (3.8–5.2) |
| **Risk category** |  |  |  |  |  |  |
| Low | 4.7 (4.0–6.0) | 5.3 (4.6–7.0) | 5.9 (5.0–7.3) | 6.8 (5.9–NR) | 7.0 (6.0–NR) | 7.4 (7.0–NR) |
| Intermediate | 2.4 (2.1–3.0) | 3.0 (2.4–4.0) | 3.7 (3.0–4.2) | 4.3 (3.8–5.0) | 4.4 (4.0–5.0) | 5.0 (4.6–6.0) |
| High | 1.6 (1.0–2.0) | 1.9 (1.4–2.5) | 2.0 (1.7–2.6) | 2.2 (1.9–3.8) | 2.2 (2.0–3.4) | 2.9 (2.1–4.6) |

Note. — NNS = number need to scan; NR = not reached.

**Supplemental Table 3**.

|  | **This study** N = 1,395 | **MESA**  **(Dzaye et al.)**  N = 3,116 |
| --- | --- | --- |
| Age, yrs. | 57 ± 10 | 57 ± 9 |
| Age categories |  |  |
| <60 yrs. | 62% | 60.5% |
| ≥60 yrs. | 38% | 39.5 |
| Females | 43% | 63% |
| Body mass index, kg/m^2^ | 23.8 ± 3.7 | 28.3 ± 5.6 |
| Waist circumference, cm | 89 ± 10 | 96 ±14 |
| Active smoking | 21% | 13% |
| Diabetes mellitus | 4.7% | 8.8% |
| Systolic blood pressure, mmHg | 125 ± 16 | 122 ± 20 |
| Diastolic blood pressure, mmHg | 78 ± 11 | 71 ± 10 |
| Serum total cholesterol, mg/dL | 221 ± 36 | 193 ± 34 |
| Serum LDL cholesterol, mg/dL | 130 ± 31 | 116 ± 30 |
| Serum HDL cholesterol, mg/dL | 67 ± 19 | 52 ± 15 |
| Serum triglycerides, mg/dL | 96 (70–143) | 107 (75–154) |
| Antihypertensive agents | 19% | 28% |
| Lipid-lowering medication | 13% | 10% |
| 10-year ASCVD risk | 7.8 ± 5.2 | 13.8 ± 13.3 |
| ASCVD risk categories |  |  |
| Low risk (<5.0%) | 34% | 31% |
| Intermediate risk (5.0%–19.9%) | 61% | 44% |
| High risk (≥20%) | 4.8% | 24% |

Note. — continuous variables are summarized as mean ± standard deviation or median and interquartile ranges (25%–75%) in parentheses, and categorical variables are summarized percentages. Data for the MESA cohort are from Dzaye O et al. (JACC Cardiovasc Imaging

2021 ;14(5):990-1002.)

LDL = low-density lipoprotein; HDL = high-density lipoprotein; ASCVD = atherosclerotic cardiovascular disease.

**Supplemental Figure 1**. Cumulative incidence of detectable coronary artery calcium stratified by sex, age, and ASCVD risk separately.


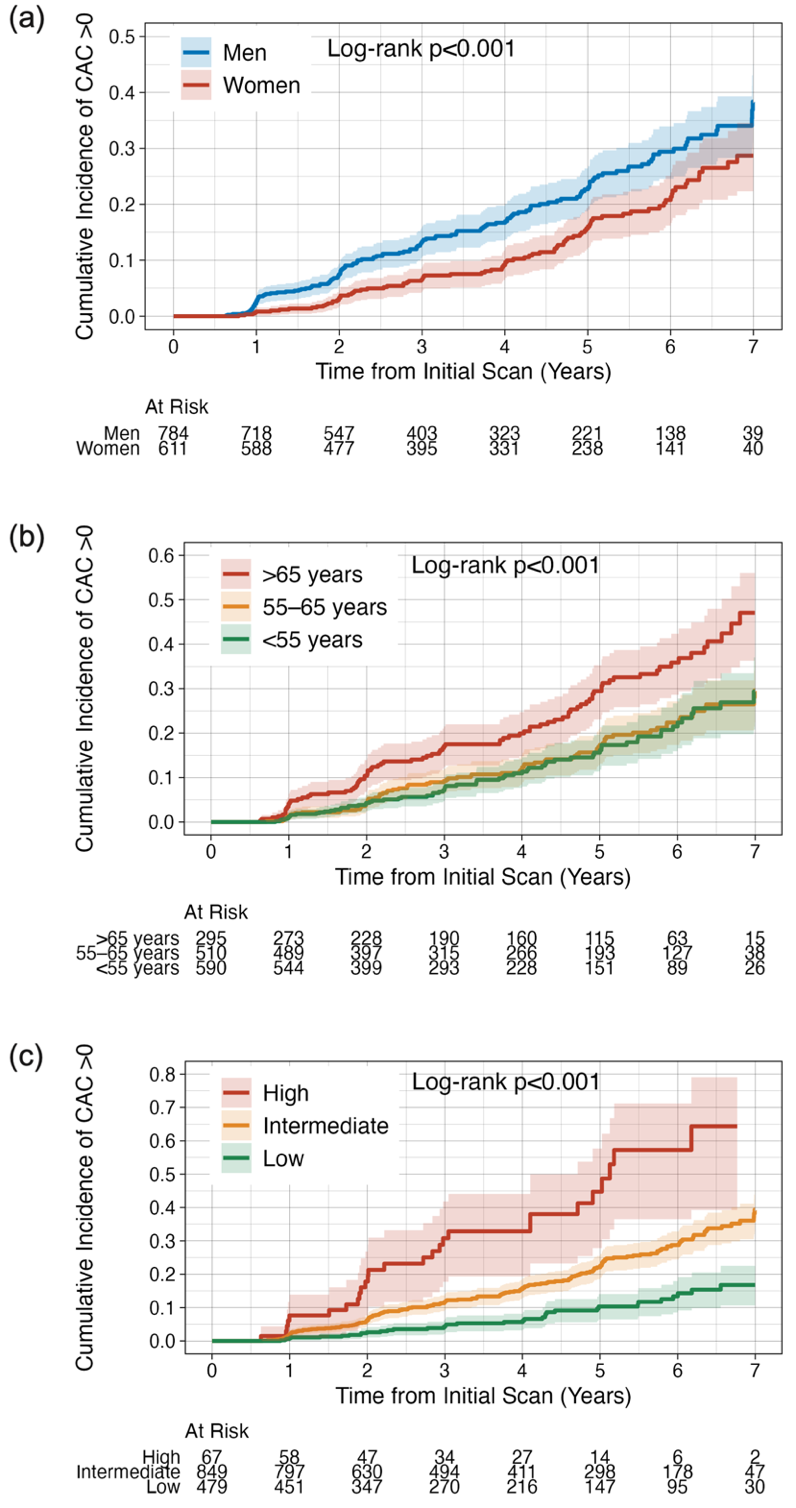


Kaplan-Meier curves show the cumulative incidence of developing detectable CAC (score >0) stratified individually by **(a)** sex, **(b)** age category (<55, 55–65, and >65 years), and **(c)** 10-year ASCVD risk category (Low <5%, Intermediate 5–19.9%, and High >20%). Shaded regions indicate 95% confidence intervals. All group comparisons were statistically significant (Log-rank *p*< 0.001).

ASCVD = atherosclerotic cardiovascular disease; CAC = coronary artery calcium.

**Supplemental Figure 2**. Estimated Time to Onset of Detectable Coronary Artery Calcium According to Age and ASCVD risk by Weibull parametric survival model.


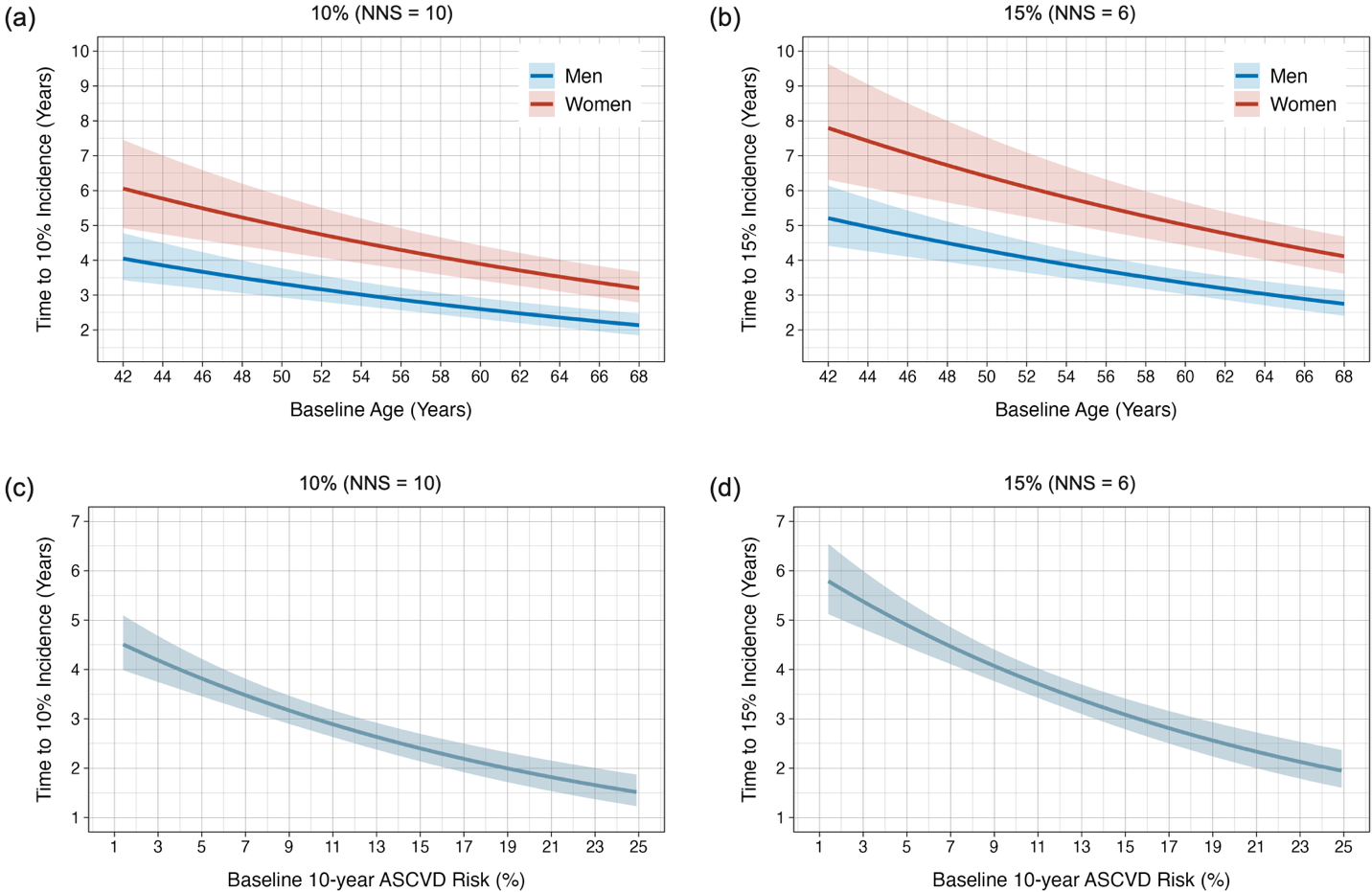


The spline curves illustrate the estimated years until the incidence of detectable CAC (score >0) reaches 10% (Number Needed to Screen [NNS] = 10; **a, c**) and 15% (NNS = 6; **b, d**). Panels **(a)** and **(b)** display estimates stratified by baseline age and sex. Panels **(c)** and **(d)** display estimates according to the baseline 10-year ASCVD risk score. Shaded regions indicate 95% confidence intervals.

ASCVD = atherosclerotic cardiovascular disease; CAC = coronary artery calcium; NNS = number need to scan.
